# Group agents, moral competence and duty-bearers: the update argument

**DOI:** 10.1007/s11098-023-01944-4

**Published:** 2023-03-24

**Authors:** Niels de Haan

**Affiliations:** 1 Department of Philosophy, Faculty of Philosophy and Education, University of Vienna, Vienna, Austria; 2 African Centre for Epistemology and Philosophy of Science, Faculty of Philosophy, University of Johannesburg, Johannesburg, South Africa

**Keywords:** Corporate duties, Collective duties, Collective moral agency, Duty-bearers, Group agency, Group duties, Moral competence, Plural subjects, We-mode groups

## Abstract

According to some collectivists, purposive groups that lack decision-making procedures such as riot mobs, friends walking together, or the pro-life lobby can be morally responsible and have moral duties. I focus on plural subject- and we-mode-collectivism. I argue that purposive groups do not qualify as duty-bearers even if they qualify as agents on either view. To qualify as a duty-bearer, an agent must be morally competent. I develop the Update Argument. An agent is morally competent only if the agent has sufficient positive and negative control over updating their goal-seeking states. Positive control involves the general ability to update one’s goal-seeking states, whereas negative control involves the absence of other agents with the capacity to arbitrarily interfere with updating one’s goal-seeking states. I argue that even if purposive groups qualify as plural subjects or we-mode group agents, these groups necessarily lack negative control over updating their goal-seeking states. This creates a cut-off point for groups as duty-bearers: Organized groups may qualify as duty-bearers, whereas purposive groups *cannot* qualify as duty-bearers.

## Introduction

Various collectivists argue that certain groups can be morally responsible in their own right. The group is morally responsible in a non-reductive manner. This is a different group-level of responsibility that does not entail or preclude individual responsibility of each individual member. For example, British Petroleum is morally blameworthy for the Deep-Water Horizon oil spill in the Gulf of Mexico. This does not necessarily mean that any member of British Petroleum is to blame for the environmental disaster, nor does it necessarily absolve any member of moral responsibility. Typically, what underlies these judgments concerning the moral responsibility of such groups is the view that these groups qualify as agents in their own right. But collectivists differ in their view which groups exactly qualify as agents.

*Procedural collectivists*, as I call them, restrict group agency to organized groups such as states, corporations or universities and claim that the mark of collective agency is rational decision-making (Collins, 2019; Copp, 2006; de Haan, 2023; French, 1984; Hess, 2014; Hindriks, 2018; Lawford-Smith, 2015; List & Pettit, 2011). These views explicate collective agency in terms of an accepted collective decision-making procedure that enables the group robustly to identify and pursue representational and motivational attitudes while satisfying desiderata of rationality.

*We-mode collectivists* expand the notion of group agency to organized groups and unorganized groups that operate in the so-called we-mode. By unorganized group I mean any group that does not have an accepted decision-making procedure and organizational structure. Basically, any group that coalesces around one or more goals may qualify as a we-mode group agent when its members think and act qua being a group member in the relevant way. I’ll focus on Raimo Tuomela’s (2007 & 2013) work and joint work of Tuomela and Pekka Mäkelä (2016 & 2020).

*Plural subject collectivists* hold that almost any instance of plural action involves a plural subject, most notably Margaret Gilbert (2014) and Hans Bernhard Schmid (2008 & 2014). They extend the notion of plural subjecthood to unorganized groups that have a single collective goal, belief, or value, as long as the members are unified in a certain way. This includes relatively large-scale groups that lack a decision-making procedure such as the pro-life lobby but also ephemeral groups such as a riot mob or two people walking together. I’ll call these unorganized groups without decision-making procedures that coalesce around one or more goals *purposive groups*. I’ll focus on Gilbert’s work.

Proponents of each of these views argue that ‘their’ group agents can be morally responsible for their actions provided certain conditions are satisfied. In this paper, I wish to bracket questions related to retrospective moral responsibility and focus on the moral duties of such groups instead. These collectivists also claim or are committed to the claim that these group agents can bear moral duties and commit moral wrongdoing (Collins, 2019; Copp, 2006; Gilbert, 2014; Gilbert & Priest, 2020, 24; Schmid 2018; Tuomela 2007, 242 & 2013, 40; Tuomela and Mäkelä 2016 & 2020). But surprisingly little attention has been paid, especially by we-mode- and plural-subject-collectivists, as to whether all ‘their’ group agents are fit to bear moral duties.^1^ My aim here is not to consider what the best and most coherent account of group agency is. Instead, my aim is to argue that purposive groups do not qualify as *moral agents*, that is, as duty-bearers in their own right, even if they qualify as group agents.

It is important to specify the target claim of this paper. I aim to show that purposive groups cannot bear what are sometimes called *corporate duties*, as the paradigmatic case involves a corporation. These are group-level duties attributed to the group agent. The group bears a duty in its own right (Collins, 2019). Corporate duties are analytically and ontically non-reducible to related member duties. This means that claims about corporate duties are not equivalent to more complicated claims about sets of individual duties at the member-level nor are they made true by facts about individual duties at the member-level (Copp, 2007; cf. Wringe 2016).^2^ In this paper, I assume the truth of the Moral Agency Principle: An agent can have moral duties only if the agent is a moral agent (cf. Wringe 2010). I take this to be a foundational principle of ethical theory. To qualify as a moral agent, the agent must have moral competence (I’ll discuss this in Sect. 2). Elsewhere I argue that organized groups can have moral competence when organized in the right way (de Haan, 2023). Here I’ll argue that purposive groups necessarily lack moral competence even if they qualify as agents. Therefore, purposive groups cannot bear corporate duties qua being a collective moral agent. In my view, there is a clear divide between organized groups, who *may* qualify as duty-bearers, and purposive groups, who *cannot* qualify as duty-bearers.

Importantly, this does not commit one to saying that all claims about the moral requirements of purposive groups are false. In the related debate concerning *collective duties*, the question is how to explicate the (intuitively correct) attribution of moral ‘oughts’ to groups that do not qualify as moral agents in certain collective contexts (e.g., see Held 1970). Some argue that these collective duties are also analytically and/or ontically non-reductive (Björnsson, 2014; Pinkert, 2014; Schwenkenbecher, 2021; Wringe, 2016). I hope to discuss this elsewhere, but, in my view, we can explicate all such claims in terms of shared duties, which are analytically and ontically reductive. Roughly, the idea is that a shared duty is nothing else but a conjunction of individual duties that are conditional on each other. Thus, the group isn’t the bearer of a group-level duty, but all relevant duties are had by individuals. The goal of this paper is to delineate the scope of the debate of collective duties more clearly by arguing for a divide between types of groups as potential duty-bearers. We must account for the moral ‘oughts’ of purposive groups without making such groups the bearer of a group-level duty, because such groups necessarily lack moral competence.

It is instructive to see that what ties organized groups together as an agent on procedural collectivism, the ‘normative glue’ if you will, is the collective commitment of members to the decision-making procedure and organizational structure. This normative glue is relatively robust, because it is a commitment to a set of *shared policies of procedure* (Bratman, 2017). For example, such procedures may specify how a group is to deliberate. At least some policies of procedure must be in place for the group to have an organizational structure and a decision-making procedure and qualify as an agent. But this robustness is not necessarily present for purposive groups, as I will explain, because the normative glue sticks to something different. This lack of robustness will become important for dealing with what I’ll call the Update Argument. Morally competent agents must have positive and negative control over updating their goal-seeking states. Roughly, positive control involves the general ability to form and update goal-seeking states, whereas negative control involves the absence of other agents with the capacity to arbitrarily interfere with forming and updating one’s goal-seeking states. I will argue that purposive groups necessarily lack negative control over updating their goal-seeking states. Therefore, purposive groups necessarily lack moral competence.

The structure is as follows. In Sect. 2, I discuss moral competence, the distinction between positive and negative control and the Update Argument. In Sect. 3, I discuss plural subject-collectivism and we-mode-collectivism. In Sect. 4, I argue that purposive groups as plural subjects and as we-mode group agents lack negative control over updating their goal-seeking states. Finally, I conclude with some reflections on the consequences of the Update Argument.

## Moral Competence and the Update Argument

To start with a relatively uncontroversial assumption, given that moral duties are generated by moral reasons, to qualify as a moral agent one must be capable of being appropriately responsive to moral reasons (cf. Fischer and Ravizza 1998). A necessary condition for qualifying as a duty-bearer is moral competence. Moral competence is the general capacity to grasp distinctively moral reasons and act in accordance with such reasons (Wallace, 1994, 2006).^3^ We may understand moral competence to involve moral understanding and control:

### Moral competence

Agent A has the general ability of moral competence if and only if (1) A has moral understanding, that is, (a) A can grasp moral reasons that possibly govern A’s actions, and (b) A can relate such reasons to A’s available evidence; and (2) A can sufficiently control her behavior accordingly in light of (1).

Moral competence enables an agent to be appropriately responsive to moral reasons. Moral competence comes in degrees, just as any other general ability, but there is a certain threshold an agent must meet to be considered sufficiently morally competent (Wallace, 2006, 125). Note that this does not mean that a morally competent agent cannot fail to be responsive to moral reasons, but the extent to which an agent is or is not responsive to moral reasons must be *up to the agent*. Of course, certain moral agents are more responsive to moral reasons than others, but all moral agents are capable of being responsive to moral reasons to a certain minimum degree in virtue of *their* moral competence. For example, a young child lacks moral understanding and therefore it is not *up to the child* whether she is responsive to moral reasons.

In what follows, I want to focus on the relevant sort of control an agent must have in order to be sufficiently morally competent. The starting point is simple: Morality cannot be unfair at its core. The fundamental principles of ethical theory mustn’t be unfair. Similar to how it would be unfair to require someone to do something if she lacked the ability to do so (Copp, 2003), it is unfair to hold someone to moral requirements when it is not *up to the agent* whether she acts in accordance with these requirements.^4^ If an agent lacks control over her behavior in light of her moral understanding, then it would be unfair to hold the agent to moral duties, because it is *not up to the agent* whether the agent is sufficiently responsive to moral reasons. Because of this unfairness, we exclude agents without moral competence from the set of duty-bearers in our moral community.

Importantly, this is not to say that morality cannot contain ‘hints’ of unfairness in some cases.^5^ For example, suppose that due to circumstantial luck someone repeatedly encounters people in need of rescue. It may be unfair *to some extent* that someone again has a duty of assistance merely because of one’s ability and presence, especially when the person must save two people instead of one because another bystander refuses to assist. However, this unfairness is outweighed by the unfairness not saving the persons in need would entail. The point is that it cannot be unfair *all-things-considered* that the agent has a duty to save the two people in need. Suppose that saving one person is fairly risk-free, but saving two people involves a high amount of risk. If this involves risk past a certain threshold, then the agent no longer has a duty *all-things-considered* to save the two persons, because it would be unfair *all-things-considered* to demand this of the agent given the high amount of risk involved.

If the core principles of morality are themselves unfair, this will generate many duty-attributions that are unfair *all-things-considered*. This would make our ethical theory incoherent. For example, it is not just *prima facie* unfair to demand a young child to respond to moral requirements they are incapable of understanding. There is not merely some consideration that points to some unfairness. This is unfair *all-things-considered* because they are themselves incapable of ensuring they are sufficiently responsive to such moral reasons. This consideration is sufficiently weighty such that we should not place them under such requirements in the first place. We mustn’t impose moral requirements upon an agent when the enabling conditions for the moral reasons that generate such moral requirements are not met. Thus, to avoid the charge of unfairness, a foundational principle of ethical theory is that only agents with moral competence qualify as moral agents, that is, as duty-bearers.

With this in mind, let’s focus on the sort of control a moral agent must have over updating her goal-seeking states in light of novel available evidence. Sometimes a moral agent must stop her intention, and sometimes she must change her intention from X to Y. I will argue that this *up-to-me-ness* entails that there are two senses of control that are both necessary for moral competence: *positive control* and *negative control*.

Suppose that an agent has an odd mental complexity, the agent can never stop intending to do X once the agent intends to do X. While doing X, it turns out that this is harming others in a manner that could not be expected up front. The agent realizes this is morally wrong, but due to her odd mental complexity she is unable to change her intention. Although her initial intention was certainly made freely, as there was no coercion involved at the start, the agent still does not seem to have sufficient control over her actions to be morally competent. The agent cannot adjust any of her behavior in light of newly discovered moral reasons. For an agent to have the right sort of control, it must be possible for the agent to stop her action midway when she discovers new reasons relevant for the current action, otherwise the agent cannot be appropriately responsive to reasons with respect to this action. If an agent can never stop her actions midway, then the agent is not sufficiently capable of being responsive to moral reasons in general.

Next, suppose the same agent has a duty to save someone. However, midway during the rescue action, the agent realizes that current action X will not succeed but that there is an alternative action Y that would save the person. This could not be expected up front. If the agent is never able to change her intentions mid-course, then the agent is not sufficiently capable of being responsive to moral reasons.

In both cases, the agent lacks *positive control* over updating her goal-seeking states in light of novel evidence. Positive control involves the agent having the ability to form intentions as a result of practical reasoning and to perform the morally required actions. We can formulate positive control as follows:

### Positive control

An agent has positive control over her goal-seeking states and actions if and only if the agent has the general ability to form and update her goal-seeking states and act accordingly in light of her moral understanding.

We can distinguish positive control from negative control. Negative control requires the robust absence of someone else’s capacity to arbitrarily interfere with forming and updating your goal-seeking states. By interference I do not mean that someone merely influences one’s goal-seeking states, but that the other agent can be decisive with respect to what your goal-seeking states are. In the absence of negative control, another agent effectively determines your goal-seeking states: either the agent forces you to intend something else or the agent allows you to intend what you intend. This sense of negative control is in the spirit of but slightly different from the republican notion of freedom as non-domination (cf. Pettit 1997 & 2001; List & Pettit 2011, 135). I am not concerned with the possible arbitrary interference with one’s *actions* or more broadly with one’s *affairs* but rather with the capacity to arbitrarily interfere with one’s *goal-seeking states*. It is not (just) that someone else can prevent you from performing an action. This is quite commonplace. For example, I can prevent you from opening the door by holding the door closed. Instead, the point is that someone else is able *at will* to prevent you from stopping an intention or forming a new intention in light of novel evidence. The absence of negative control implies the presence of some form of manipulation.

To illustrate the importance of negative control for moral competence, consider the following case.

### Manipulation

John can initially freely form any intention at will. However, an evil neuroscientist has implanted a device in John’s brain, which enables him to prevent John from updating *any* of his intentions. Whenever John attempts to stop or change his intention, the evil neuroscientist can intervene. John intends to go for a boat ride. When inspecting the boat, he notices the boat has an oil leak. John attempts to stop intending to go for a boat ride, but the evil scientist prevents him from stopping his intention. During the boat ride, a large quantity of oil leaks into the lake endangering the local fish population. Many fish will die within the hour unless they are rescued. Distraught with what happened, John wishes to do what he can to save the fish population. At first, he sees no other option but to catch as many fish as possible with another fishing boat with the hopes of releasing them in a nearby pond. This won’t save all the fish, but it seems John’s best option. While retrieving the other fishing boat, John finds a pump system that could clean up the entire oil spill. John attempts to change his intention to install the pump system instead of catching as many fish as possible. The evil scientist, relishing in his power, decides to let John do so. John installs the pump system and cleans up the oil spill. The fish population is saved.

Is John sufficiently morally competent such that it would be fair to hold John to moral requirements? I don’t think so. John has moral understanding, but he does not have sufficient control over his behavior. John realizes he is about to violate a duty not to harm the environment when using the boat, because it has an oil leak. However, the evil scientist prevents him from stopping his intention to go for a boat ride, which is why John causes the disaster. Next, realizing he ought to save the fish population, John starts with an initial rescue attempt. During his preparation, he realizes that there is a better way. The evil neuroscientist *allows* John to change his intention. While John ultimately succeeds in saving the entire fish population, this success was in an important way not *up to him.* Whether or not the evil neuroscientist actually interferes is irrelevant. What matters is that he *can*. The point is not that John chose to do something that the manipulator would have forced John to do something anyway (cf. Frankfurt 1969). The point is rather that the manipulator has the capacity to arbitrarily interfere with *all* of John’s attempts to update his goal-seeking states. When another agent can prevent you from stopping or changing *any* intention at will, you do not have sufficient control over your goal-seeking states, because it is no longer *up to you* whether you are sufficiently responsive to reasons (see also Wedgwood 2013, 77). Your reason responsiveness is to a very large degree determined by another agent’s responsiveness to these reasons concerning *your* behavior. Whether you will in effect discharge any of your duties is essentially not up to you. Therefore, it is unfair to hold John to any moral duties, at least as long as the evil neuroscientist can manipulate him, because whether or not he is sufficiently responsive to moral reasons is not up to him.

We can formulate negative control as follows:

### Negative control

An agent has full negative control over her goal-seeking states if and only if no other agent has the capacity to decisively arbitrarily interfere with forming and updating her goal-seeking states.

Negative control comes in degrees. If a manipulator has the capacity to arbitrarily interfere with *all* your goal-seeking states, then the degree of negative control is zero.^6^ What I am arguing is that if someone else has decisive control over updating your goal-seeking states, then this is a severe impediment to your moral competence, because the degree to which you are responsive to moral reasons is not up to you, but up to someone else. It is not up to you whether you do what is morally right.

If this is correct, then I think the following argument follows. I call it *the Update Argument*.

*Premise 1*. All duty-bearers are morally competent, and an agent is morally competent only if the agent has moral understanding and can control her behavior in light of her moral understanding.

*Premise 2*. An agent can control her behavior in light of her moral understanding only if the agent has a sufficient degree of positive and negative control over updating her goal-seeking states in light of her moral understanding.^7^

*Conclusion*. Any agent without a sufficient degree of positive and negative control over updating their goal-seeking states lacks moral competence and does not qualify as a duty-bearer.

Next, I turn to the two different variants of collectivism. On either view, do purposive groups qualify as duty-bearers?

## Plural Subject Collectivism and We-Mode Collectivism

In this section, I’ll set out plural subject and we-mode collectivism. In Sect. 4, I’ll apply the Update Argument to both accounts.

*Plural subject collectivists* hold that almost any instance of plural action involves a plural subject, provided the group is unified in the right way. The group is the *subject* of an intention. Given that plural actions merely require the participation of two or more individuals acting in pursuit of one and the same token goal (Schmid, 2008, 25), such views expand the concept of intentional subjects considerably. I’ll focus on Gilbert’s view.

Gilbert argues that a group of agents constitutes a collective agent if and only if they are jointly committed to doing something as a body. The agents collectively intend to perform action A (e.g., walking together) if and only if they are jointly committed to intend as a body to perform A (Gilbert, 2014, 70). To intend as a body essentially is together to constitute, as far as possible, a single body that intends to do that thing (Gilbert, 2014, 63). A joint commitment is a commitment of all members of the group, it is not a conjunction of personal commitments. Joint commitments are formed when ‘a pool of wills is formed’, that is, when each agent expresses her willingness to do her part in conditions of common knowledge.

Joint commitments are inherently normative. Obligations and rights inhere in any joint commitment, although these obligations and rights are not necessarily moral. This is important to emphasize, because it is possible that a plural subject is jointly committed to do something immoral (e.g., robbing a bank). Each party is answerable to one another for violations of the joint commitment, meaning each has the standing to rebuke. This is entailed by each agent having a directed (non-moral) obligation toward the other agents to adhere to the joint commitment and in turn each party has a correlated right to claim each party’s conformity to the joint commitment (Gilbert, 2014, 108–109). Call this the Obligation Criterion. A joint commitment that results from entry into an agreement retains its normative force until it is rescinded, but, failing special circumstances, this can only be done together and not unilaterally (Gilbert, 2014, 106). Call this the Concurrence Criterion. Even if the plural subject is doing something immoral, these conditions hold.

Both the Obligation and Concurrence Criterion argue against the sufficiency to account for collective intentions in terms of correlative personal intentions (Gilbert, 2014, 112, cf. Bratman 2014, 116). A joint commitment (and thereby a collective intention) may persist even when participating agents no longer have relevant personal intentions, because unless the joint commitment is (universally) rescinded, the agents still owe their conformity to the joint commitment to the other parties. Because of this, statements such as ‘we intend to do A’ cannot readily be reduced to a set of statements with singular subjects (we both intend to do A), rather the reference of ‘we’ is a plural subject (we *collectively* intend to do A). Thus, in Gilbert’s view it is the normativity of a joint commitment what makes collective intentions irreducible to individual intentions. This will be important to keep in mind when we turn to a problematic consequence of the Concurrence Criterion in Sect. 4.

Building upon her analysis of the plural subject, Gilbert argues that plural subjects can be collectively morally responsible. Gilbert claims that if ‘one freely engages in an action that, as one knows, violates a moral requirement’, then blameworthiness is instantiated at the collective level (2014, 62). We must see whether the plural subject has been subject to external pressure and coercion or whether it makes sense to say that a given collective was free to act as it did (2014, 73). Likewise, in joint work, Gilbert and Maura Priest state that ‘one who performs an action is morally to blame for doing so if and only if the following three conditions are met: the action was morally wrong, all else being equal; all else was equal: in particular, one was not coerced into performing the action, and one knew, or was at least in a position to know that the first two conditions were fulfilled’ (Gilbert & Priest, 2020, 24). Gilbert and Priest claim that plural subjects can be morally blameworthy for performing actions that are morally wrong. Moral wrongdoing entails the violation of a moral requirement. Although Gilbert does not say anything about the exact nature of the moral requirements of plural subjects, given that she does think that there is moral responsibility at the collective level, the most straightforward interpretation is that Gilbert thinks that the moral requirements of the plural subjects are likewise at the collective level. Hence, Gilbert seems committed to the claim that plural subjects can have group-level moral duties and that plural subjects necessarily have moral competence.^8^

Next, let’s turn to we-mode collectivism. *We-mode collectivists* hold that when the members of a group act jointly in the we-mode, conceptually we can speak of them acting as a group. It is important to distinguish between two types of groups: organized groups and we-mode groups. Both these groups can be regarded as functional group agents that have *extrinsic* mental states, states collectively attributed to them (primarily by their members), as opposed to *intrinsic* mental states and phenomenal features that a regular person typically has on biological and psychological grounds (Tuomela & Mäkelä, 2016, 299–300). Technically, an organized group can be a ‘we-mode group’ or an ‘I-mode group’ (Tuomela, 2013, 88). Here, I am interested in spelling out the role of commitment to the ethos and how purposive we-mode groups are different from organized groups in this respect.

One and the same intention (in terms of content) can be had in different modes, according to Tuomela, namely in the collectivistic we-mode or in the individualistic I-mode. To think and act in the we-mode is to think and act fully as a group member. Members of a we-mode group act for group-centered motives and reasons. They reason from the group’s point of view. The group intends to achieve a goal if and only if its members intend jointly, as a group, to achieve it. Consider “We will do X together as a group”. ‘The intentional agent of the intention is “we” (namely, the group members forming a non- distributive “we”), and its content is the members’ jointly intentionally performed joint action’ (Tuomela & Mäkelä, 2016, 304). We-intentions are basically slices of a joint intention. And the satisfaction of the we-intention is *for* the group. If a person truly acts *on* his we-mode intention, he acts qua group member.

To be precise, consider Tuomela’s summary account of we-intention:

### (WI)

Member A_i_ of group g we-intends in the we-mode to perform X together as a group with the other members if and only if, given that the we-mode criteria for intending are satisfied (in the sense of WMI of Tuomela 2013, 68)^9^,

(i) A_i_ intends to participate in the members’ (“our”) doing X together and to do his part of X as his part of X;
(ii) A_i_ truly believes that the group members (including himself) collectively accept “We will do X together as a group” in that context and that thus a joint intention to perform X jointly exists between the participants, and this fact is his main justificatory reason for (i);
(iii) A_i_ has a true belief to the effect that the joint action opportunities for an intentional performance of X with some likelihood will obtain, especially that a right number of the full-fledged and adequately informed members of g, as required for the performance of X, will at least with a non-negligible probability perform their parts of X, which under normal conditions will result in an intentional joint performance of X by the participants;
(iv) A_i_ truly believes that there is (or will be) a mutual true belief among the participating members of g (or at least among those participants who perform their parts of X intentionally as their parts of X) to the effect that the joint action opportunities for an intentional performance of X will obtain (or at least will obtain with a non-negligible probability);
(v) (i) and (ii) are in part true because of (iii) and (iv). (Tuomela, 2017, 31)

In virtue of being collectively accepted by the group members qua group members, the we-mode we-intention satisfies the three central criteria involved in the we-mode: group reason, the collectivity condition, and collective commitment.

Clause (ii) in conjunction with (iii) and (iv) gives a group-based reason for the members to participate in the joint intention and thus to form their part-performance intention and normally to perform their action (Tuomela, 2017, 31). It is important here to distinguish between a *group agent’s reasons*, which are reasons that a group agent has for performing its actions, and the *group-based reasons* of its members for participating in the group’s activities (Tuomela, 2013, 98). An ideal we-moder thinks and acts only for authoritative, *group-based*, uniformly motivating reasons, regardless of whether they conflict with individual motives.

According to the *collectivity condition*, it is necessarily the case that the goal is satisfied for the we-mode group only if it is satisfied for all members. The members perform X *for the group*, which means that the members are necessarily ‘in the same boat’ with respect to X.

Basically, *collective commitment* has two roles. First, collective commitment is the normative glue that binds the members of a we-mode group together in relation to the group’s ethos (Tuomela, 2013, 82). The ethos is made up by the group’s constitutive goals, interests, values, beliefs, practices, and/or norms (Tuomela, 2007, 32). After forming the group ethos, the members collectively commit themselves to acting in accordance with it (Tuomela & Mäkelä, 2016, 301). As a result, individual members are not allowed (in a non-moral sense) to act against the ethos, and the ethos can only be changed or dropped via collective acceptance. Collective commitment to the ethos provides the foundation for the unity and identity of the group (Tuomela, 2007, 5; 2007, 35). The group cannot be a unit unless it forms a strong “we”, which requires the members to be attached to the identity-expressing ethos by collective commitment (2007, 36). Collective commitment to the ethos is ‘the central ingredient that accounts for the stability and robustness of group life’ (Tuomela, 2013, 44).

Second, the collective commitment of members to the satisfaction of the group’s ethos and performing their parts, serves to give joint authority to the group members to pursue ethos-related action (Tuomela, 2007, 5; 2013, 38–46). Basically, collective commitment to the ethos gives an authoritative group-based reason for the members of a we-mode group to act. This may conflict with private goals, beliefs, and so on (Tuomela, 2013, 256). As Tuomela puts it: ‘Collective commitment gives the “glue” that normatively (not necessarily morally) binds the members together in accordance with the group reason in a stable way’ (2013, 246).

The ethos plays a crucial role in tying the we-mode group together:

(WMG) A collective g consisting of some persons (or in the normatively structured case, position-holders) is a we-mode social group (if and) only if.

1. g has accepted a certain ethos, E, as a group for itself and is committed to it;
2. every member of g group-normatively ought to accept E as a group member (and accordingly to be committed to it as a group member), at least in part because the group has accepted E as its ethos;
3. it is a mutual belief in the group that (1) and (2). (2013, 27)

Note that the normative glue that provides the unity of purposive we-mode groups sticks to the ethos rather than any set of procedures. Members only give up authority with respect to pursuing the group ethos. This allows for a wider range of group agents, as not all we-mode groups necessarily have a set of shared policies of procedure, but the continued existence of such we-mode groups depends heavily on what the ethos exactly entails. The ethos can be a large set of values, goals and beliefs (e.g., the pro-life lobby) or just a single token goal (e.g., going for a walk together). I will return to the importance of the ethos in Sect. 4, as this plays an important role for the Update Argument.

Building upon Tuomela’s analysis of we-mode group agency, Tuomela and Mäkelä (2016) argue that we-mode groups can be blameworthy for their actions, which they call group responsibility (see also Tuomela 2007, Ch.10; Tuomela and Mäkelä 2020, 74 − 6). A well-functioning we-mode group generally can qualify as a bearer of and be fit for responsibility (Tuomela & Mäkelä, 2016, 301). Group responsibility is ascribed *jointly* to members of the group acting qua members of the group: the group members are *together* and *interdependently* responsible for the action (Tuomela & Mäkelä, 2020, 66). Note that, ultimately, the ‘’truthmaker’’ of a collective attribution of moral responsibility is the responsibility borne by individuals because this is something members have *jointly* (Tuomela & Mäkelä, 2020, 65). But the actions and responsibility of a we-mode group agent do not reduce to the individual level. They say: ‘A group’s responsibility, of course, is connected to its members’ collective responsibility at least when they act as group members, but […] it still often differs from the members’ full-blown joint responsibility’ (Tuomela & Mäkelä, 2016, 300). They claim that like group attitudes, group responsibility is non-redundant and irreducible to the member-level (Tuomela & Mäkelä, 2016, 307). Thus, Tuomela and Mäkelä hold that we-mode group agents are subject to collectivistic attributions of actions and responsibility.

Basically, they claim that if a we-mode group g is blameworthy for group action X relative to a deontically relevant normative standard S, then, prior to the action, group *g* ought to have seen to it that Y or brought about Y, where Y is an action or outcome of action that is incompatible with X (Tuomela & Mäkelä, 2016, 315).^10^ There are other conditions for group responsibility, but let’s focus on the ‘ought’ attributed to the we-mode group. This ‘ought’ can be a ‘moral ought’. For example, when the group has promised to do something to some party (Tuomela, 2007, 240). Thus, they claim we-mode groups can be the subjects of moral ‘oughts’.^11^ In order for a we-mode group to have a moral ought, that is, a moral duty, the agent must be morally competent. Hence, I take it that Tuomela and Mäkelä are committed to the claim that we-mode groups, including purposive groups, are morally competent.

When discussing the control condition for moral responsibility, Tuomela and Mäkelä note that we-mode groups (and structured groups) do not have direct control over a body that could move, as collectivities only act through their members’ actions, but these groups have indirect control. Indirect control amounts to ‘filtering for what is in accordance with the group’s ethos and excluding other possibilities’ (Tuomela & Mäkelä, 2016, 308). The group agent plans for what its members must bring about via its ethos, and the group monitors and controls that the group members carry it out when the circumstances are feasible. Because members who identify with the group are disposed to act in accordance with the ethos, the ethos plays a crucial role in the motivation of members to perform specific actions conducive to the group’s aims. Via this psychological mechanism, the group can be said to intermediately cause the group’s relevant action. Note that collective commitment to the ethos plays a crucial role for indirect control. I’ll return to this shortly. (Tuomela & Mäkelä, 2016, 308)

Thus, both Gilbert’s and Tuomela and Mäkelä’s view seem to entail that purposive groups can have group-level duties, and thus that purposive groups are morally competent agents. In what follows, I’ll argue that purposive groups necessarily lack moral competence on either account.

The problem is not that these groups lack moral understanding. Elsewhere, I argue that an organized group can have moral understanding in virtue of their members’ moral understanding (de Haan, 2023; cf. Hindriks 2018).^12^ If the group’s members are able to grasp moral reasons that govern the possible actions of the group and the members are able to put forward these moral considerations into the group’s decision-making process, then the organized group has moral understanding, provided the organized group can adequately relate such reasons to the group’s available evidence. In larger groups, the access to the group’s available evidence may be dispersed such that not every member has access to all the group’s available evidence. I think something similar holds for the moral understanding of purposive groups.

Instead, the problem lies with control.

## Lack of Negative Control over Updating Group Intentions

In this section, I’ll argue that purposive groups as plural subjects and as we-mode group agents necessarily lack moral competence, because they lack negative control over updating their group intentions.

- Plural Subject Collectivism.

Gilbert is right of course that if an action was coerced then the agent (typically) is not responsible for the action. And if an agent could never act freely, then this would disqualify the group as a duty-bearer. However, this capacity for freely acting does not equal the stronger sense of control that is required for moral competence. There is a rather odd consequence of Gilbert’s account that, to the best of my knowledge, surprisingly has gone largely unnoticed.^13^

Suppose agents X, Y, and Z are jointly committed to action A by way of express agreement. After establishing the joint commitment, it becomes clear that A-ing is morally wrong. This could not be foreseen up front. If the genesis of the basic joint commitment is an agreement, absent any special background understandings, then the concurrence of all parties is needed for rescinding the foundational joint commitment, where rescission is a deliberate, explicit cancellation of the joint commitment (Gilbert, 2009, 182). Thus, given the Concurrence Criterion, in order to stop collectively intending to do A, every single member must agree to do so or at least waive their right to claim conformity to the action specified in the joint commitment. Otherwise, the normative force of the joint commitment remains in play and therefore the collective intention on Gilbert’s view. This raises the question to what extent these plural subjects can be said to have sufficient control over updating their goal-seeking states.

Let’s consider positive control first. Plausibly, group-level abilities supervene on the individual abilities of members (Collins, 2019). What a group has the ability to do does not depend on what members are *disposed* to do. For example, we both may be disposed to walk together through Central Park above anywhere else, but that doesn’t mean we aren’t *able* to walk together on Fifth Avenue. Likewise, given that each member can waive their conformity to the joint commitment, clearly, there is a sense in which the plural subject *can* stop its intention, even if members are not likely to do so. Of course, there may be circumstances in which a plural subject cannot be said to have the relevant ability to form an intention, because the formation of the joint commitment itself is impeded. But this does not show that there is necessarily something problematic about purposive groups as duty-bearers, as one may simply deny that the plural subject in that case has a duty. The problem is not that the plural subject lacks positive control.

Yet, there seems something amiss. The problem is that the plural subject lacks negative control. A plural subject’s initial intention may have been freely formed, but if the plural subject cannot alter the course of action without every member concurring, then the plural subject cannot be said to have negative control over updating its goal-seeking states, because it is dependent on the concurrence of every single member for stopping or changing its collective intention. There is a lack of group-level negative control because each member has the capacity to prevent the plural subject from stopping or changing its intention. This means that it is decidedly not *up to the plural subject* whether it stops A-ing or whether it changes its intention from A to B (where B is what is morally required). Note that in Manipulation the worry was the presence of one manipulator, but here in effect *every* member of the plural subject is a manipulator. Every member has the power to prevent the plural subject from stopping their intention to A or from changing its intention from A to B. This means that it is decidedly not solely *up to the plural subject* whether they stop their wrongdoing or whether they change the course of action to do what is morally right.

Organized groups do not have this control problem on Gilbert’s view. Gilbert distinguishes between basic and non-basic joint commitments. A non-basic joint commitment is created for the members of a population by some party who has been authorized to do so by means of a basic joint commitment of those members (2014, 218). The basic joint commitment authorizes a given person or body to create new joint commitments for the parties, which can be done without knowledge of all parties (2014, 398). For example, all members of a corporation accept as a body that the board and sub-committees are to decide what the group’s intentions are. Here, the other members do not have the capacity to arbitrarily interfere, because they have relinquished any such related claim-rights by committing to the basic joint commitment that authorizes the board to make non-basic joint commitments. Thus, organized groups have group-level negative control over updating its goal-seeking states in virtue of its accepted decision-making procedure.

However, plural subjects without non-basic joint commitments do not have group-level negative control over updating their goal-seeking states once the joint commitment is in place. The point is that when the plural subject tries to stop or change its goal-seeking state, members have the capacity to prevent the plural subject from succeeding. What makes this capacity to interfere arbitrary is that no member agreed to these members having this kind of authority over the goal-seeking states of the plural subject. Similar to how John’s reason-responsiveness is dictated by the reason-responsiveness of the evil neuroscientist in Manipulation, here the reason-responsiveness of the plural subject is dictated by the reason-responsiveness of a few members without the rest of the members having agreed to this. These members have this kind of authority simply because of the way the plural subject is constituted. Whenever a purposive group exercises its positive control over updating a group intention, there will always be a set of members that at the same time have the capacity to arbitrarily decisively interfere with updating its goal-seeking state. Thus, it is in virtue of how purposive groups as plural subjects are constituted that they lack negative control over stopping or changing their goal-seeking states. In order to acquire such negative control, they would have to become a different type of plural subject entirely, namely an organized group. Because only organized groups have the requisite power and authority to stop or redirect goal-seeking states without the concurrence of all members.

This means the Update Argument is applicable to purposive groups as plural subjects. Purposive groups as plural subjects lack negative control over stopping and changing all their goal-seeking states. Any agent without a sufficient degree of negative control over updating their goal-seeking states lacks moral competence and fails to qualify as a moral agent. Therefore, purposive groups as plural subjects lack moral competence, and do not qualify as duty-bearers.

Some may wonder whether this argument holds for all purposive groups. The lack of group-level negative control arises due to the Concurrence Criterion, which is pivotal for Gilbert’s claims concerning the irreducibility of the group intention to personal intentions. It’s hard to see how this does not hold on Gilbert’s view for small-scale plural subjects (e.g., two people walking together). Gilbert explicitly relies on this condition in her defense against Michael Bratman’s arguments for a reductive account of shared action (Gilbert, 2009, 17; cf. Bratman 1993 & 2014). But perhaps in larger plural subjects without non-basic joint commitments, the Concurrence Criterion should be weakened to some extent. One might argue that individual refusals to stop a group intention doesn’t dictate what the entire plural subject intends to do. One could try to reformulate the Concurrence Criterion for large-scale plural subjects such that when a majority of the members relinquishes their right to conformity, the normative force of the entire joint commitment is rescinded. In terms of positive control, the group-level ability to stop a group intention is instantiated when a majority of members exercise their individual ability to relinquish their right to conformity.

However, this does not solve the problem. Suppose we have a plural subject that is unified around a single joint commitment. It seems odd to think that the plural subject is entirely disintegrated when a minority but nonetheless substantial number of members continue to be jointly committed to the initial goal-seeking state. Instead, it seems a majority of members have left the plural subject (without necessarily thereby constituting a new plural subject) and the remaining members still constitute the initial plural subject. For example, suppose a riot mob qualifies as a plural subject. If the majority of the mob that is rioting in the city disperses, say 1000 members, but a minority yet still substantial number remains committed to rioting in the city as a body, say 500 members, the plural subject doesn’t disintegrate, instead the plural subject has simply shrunk in size. After all, we readily accept that groups can undergo membership changes. The same riot mob is still there, rioting in the city and destroying property, it’s just smaller. The other 1000 members have left the plural subject, but they have not prevented the plural subject from retaining the initial group intention. Thus, *the plural subject* continues to intend what it intends.

A similar problem arises when the majority of members try to change their group intention. Suppose 1000 members want to change the group intention to cleaning up their mess instead. However, 500 members stick to the initial joint commitment. If the group’s identity clusters around the initial joint commitment that characterizes the group, which in this case is a joint commitment to rioting in the city, then the original plural subject has not changed its group intention. Instead, the 1000 previous members have created a *new* plural subject that is now cleaning up the mess.

The problem with negative control reappears. The problem is that any (substantial) minority of members within the plural subject have the capacity to arbitrarily interfere with the plural subject stopping or changing their group intention. They have this capacity simply because of the way the plural subject is constituted via the initial joint commitment and how the group’s identity over time is attached to the initial joint commitment. Again, this means that each minority within a large-scale purposive group in effect is akin to an external manipulator. I’ll return to group identity over time in more detail shortly, as Tuomela explicitly discusses group identity over time and a similar problem arises for we-mode collectivism.^14^

Some may object instead that this argument ignores the interpersonal dynamics within plural subjects, as other members may try to prevent these members in various ways from exercising this capacity.^15^ For example, they may place pressure on existing members by threatening to expel or shun them from the group unless they relinquish their right. Of course, such pressure may make it less likely that these members exercise their capacity to arbitrarily interfere, and change what these members generally are disposed to do. But while it may be less likely that these members prevent the plural subject from stopping or changing its intention, this does not remove these members’ *ability* to arbitrarily interfere. The members still have this right to claim conformity to the joint commitment, and these members *can* hold on to this right. Thus, the capacity to arbitrarily interfere remains despite such internal pressure.

Next, I’ll show that a similar problem arises for purposive groups as we-mode group agents.

b. We-Mode Collectivism.

Interestingly, on Tuomela’s view, a member also typically cannot unilaterally rescind her commitment to the ethos without the others’ permission, because she has given up part of her authority to act to the group and needs the others’ permission to get it back (2013, 43). But Tuomela holds that a *pro tanto* group-based reason can be outweighed by moral reasons (2013, 119). According to Tuomela, ‘if the harm ensuing from a member’s abandoning the commitment is small, merely *informing* the group may suffice’ (2013, 43, emphasis added). This means that members do not have a continued entitlement to the conformity of other members unless they relinquish that right. Hence, for small-scale ephemeral purposive groups, such as three people walking together, when it becomes apparent that the group action is morally wrong, this commitment need not be unilaterally rescinded by each member. Given that Tuomela does not rely on the Concurrence Criterion, one might think that the Update Argument does not apply to his account.

But as discussed earlier for plural subjects, the problem is also generated by the strong dependency of the group’s identity over time on the initial collective commitment that characterizes the purposive group. Tuomela’s purposive we-mode groups run into the same problem. While Tuomela and Mäkelä are right that the ethos amounts to some group-level indirect control by filtering for what is done in accordance with the ethos, this control is not yet sufficient to qualify as a duty-bearer. The problem is clearest for small-scale ephemeral purposive groups whose ethos consists of a single token-goal. The members have only given up their authority with respect to that ethos. Hence, if the group is to change its intention, the members are not necessarily committed to this new ethos. This gives rise to a control problem that relates to the group’s identity. Consider what Tuomela says:

Without a substantial amount of collective commitment to the ethos, the group risks losing its identity (or part of it) and falling apart into an aggregate of independent actors furthering the same ethos. Collective commitment functions to guarantee that the identity stays the same, or becomes what the group wants it to become, and such commitment serves to unite the members around that identity. (Tuomela 2007, 36)

Thus, in Tuomela’s view, the commitment to the ethos is essential for the unity and continued existence of the we-mode group agent. This doesn’t mean that the we-mode group *cannot* change their group intention, they certainly can, namely when all members exercise their relevant individual abilities. Again, as with Gilbert, the problem is not positive control, but negative control.

In small-scale purposive groups, because members are only collectively committed to the current ethos, when a new goal is to be pursued, the members must again collectively commit themselves to this goal. The collective commitment that ties the group together as a unit is only there with respect to the single-token-goal. The group’s ethos simply is nothing more than this goal. And this makes sense in a way. Members only give up their autonomy with respect to the ethos. It would be odd if the group could simply change its ethos and every member is nonetheless committed to continue to be part of this group. However, in order for the purposive we-mode group to remain the same group when updating its group intention, members must again collectively accept this new ethos, otherwise the group falls apart into an aggregate of independent actors. But this means a minority of members can prevent *this* we-mode group from updating their intention. What we see is that with the sort of group agency at play for purposive groups, the unity provided by the commitment is not robust enough for the group to qualify as a duty-bearer, because it in effect makes members of the group akin to the evil neuroscientist in Manipulation. These members can prevent the group from making any changes to the ethos, because without their renewed commitment the we-mode group will disintegrate into an aggregate of independent actors. Therefore, these single-token-goal purposive groups do not have group-level negative control over updating their goal-seeking states.

For the group agent to have the authority to change its current goal-seeking state to another goal-seeking state *without* the renewal of commitment from members, the members must have granted this authority to the group agent. But for the group agent to acquire such authority, this essentially involves members agreeing to a particular kind of decision-making structure that allows the group agent to make changes to the ethos. But this would effectively turn the purposive group into an organized group. In hierarchical groups, the authorized leaders have the power to form an intention that gives the ground for the nonoperative members’ assumed collective acceptance of this intention (Tuomela, 2013, 112). Because organized groups do have the authority to redirect the goal-seeking states without renewed commitment from members, organized groups do have negative control over updating their goal-seeking states.

But what about larger purposive groups with a more substantive ethos one might ask. These groups seem more robust in their unity, as their ethos is made up of more than just one goal. To see why this is still a problem, let’s turn to Tuomela’s account of group identity over time:

### Group Identity over Time

g at t_1_ is (largely) the same as g’ (or g is largely ‘‘collectively identical’’ with g’) at t_2_ if and only if.

(i) the content of the ethos of g’ at t_2_ is obtained from that of g in terms of a family-resemblance transformation based on collective acceptance;
(ii) g’ is a causal descendant of g;
(iii) the members of g’ collectively accept in effect (i) and (ii) as true;
(iv) it is a mutual belief among the members of g’ that (iii). (Tuomela, 2007, 148, slightly modified).^16^

This account is incomplete. As it stands, it cannot account for cases where a group branches into two groups. In condition (i) it is left unspecified whose collective acceptance is required. The best interpretation I think is that this is by *all members of g’*.^17^ In cases where all members of g collectively accept the transformation of the ethos, all members of g’ are the same, and there is no problem. But in cases of secession and splitting, where members of the initial group branch into two groups, the account runs into difficulties.

Let’s assume that the pro-life lobby qualifies as a purposive we-mode group. Suppose that a minority of the members of the pro-life lobby decides to stop their lobby activities for pro-life. They do not give up the underlying values and beliefs concerning abortion, but they no longer think lobbying is the best way to promote those values, instead they focus on infiltrating the educational system. They collectively accept this as part of the new ethos. The content of the ethos of ‘pro-life educators’ is obtained in terms of family resemblance transformation; they are a causal descendent of the pro-life lobby; and let’s assume that the members collectively accept this and that this is a mutual belief. The other group members of the pro-life lobby remain collectively committed to the old ethos. According to Tuomela’s account, *both* groups are now identical to the initial group that we called the pro-life lobby. Clearly this cannot be right. After all, only one group is actually lobbying for pro-life!

To address this issue, we need a contextual condition that resembles what Robert Nozick (1981) calls the closest continuer. Whether or not a group is the continuation of the previous group depends on what other groups there actually are:

### Closest Continuer

At t_2_, there is no g’’ that is a causal descendant of g that stands in a closer relation to g at t_1_ than g’ at t_2_.

How do we spell out what this closer relation exactly means? Nozick’s example of the Vienna Circle is instructive (1981, 32). Suppose that after the Vienna Circle was driven from Austria by the Nazi regime, three members, including Hans Reichenbach, landed in Istanbul and continued to meet to discuss philosophy and adhere to the same philosophical program. They believed all of the others were dead. After the war, they learn that nine members of the Circle had fled to the United States, and continued meeting there in the same fashion. Nozick suggests that the group in the US is the closest continuer, whereas the group in Istanbul is an offshoot. If, however, the group in the US did not exist, the group in Istanbul would be the real Vienna Circle because they are the closest continuer.

I’m not sure Nozick is right that the number of members is necessarily the deciding feature, but Nozick is certainly right that physical continuity matters for identity-over-time when the groups’ ethe are identical. For example, suppose that among the members of the group in the US were Rudolf Carnap and Kurt Gödel. It seems right to say that the real Vienna Circle met in the US, whereas the group in Istanbul was merely an offshoot, even though both groups have the same ethos. However, given that different groups can have the exact same set of members, physical continuity in terms of membership clearly cannot be the only deciding factor.

As important, if not more, is the groups’ ethe. If there is a g’’ whose ethos is the exact same as g, and g’’ is a causal descendent of g, then it stands to reason that g’’ *is* g, and g’ is not g when g’ has an ethos that differs from g. Suppose the pro-life lobby indeed splits into two groups. Even if some of the now pro-life educators were highly influential members within the initial group, it still seems correct to say that the group that remains committed to lobbying for pro-life simply *is* the pro-life lobby, because this was the identity defining feature of the initial group to which the members were collectively committed. The pro-life educators have simply split away from the pro-life lobby.

Of course, for groups with a sufficiently rich ethos where there is an inner conflict about the direction of the group, it may be up for debate which group is identical to the original group. The ideological battle will partly be about which group most preserves the initial ethos or encapsulates best the set of aims, values and beliefs of the original group. I hope to set out a more detailed account of group-identity-over-time elsewhere, but this is not my endeavor here. The point is that when there is a group with no change in the initial ethos and a branching group with a revised ethos (and all else being equal), the group with the same ethos stands in the closest relation to the initial group. And if conditions (i) to (iv) are also satisfied, then the group with the same ethos simply is the original group. To accommodate the possibility of branching, any adequate account of group identity over time needs (a condition resembling) the Closest Continuer condition.

Next, with a better understanding of group identity over time, we can apply the Update Argument to large-scale purposive we-mode groups. Suppose the purposive group morally ought to do something that does not fall under their current ethos. In a simplistic way, the new ethos could involve the adoption of a new goal. Or perhaps in a more substantive way, the question may become whether the group should value something other than it currently values, where that new value would subsume the goal. In both cases, the ethos must be amended. This new ethos must be collectively accepted, and again branching becomes possible. For example, the pro-life lobby morally ought to stop lobbying for pro-life and support women’s rights instead. The problem is similar to the example of the riot mob as a plural subject I discussed earlier: when a set of members remain committed to the earlier ethos of the group, then this remaining part simply *is* the we-mode group at t_2_. The other group has constituted a *new* we-mode group. But the initial we-mode group has not changed its goal-seeking state. Again, the point is not that the we-mode group *cannot* change their intention, it certainly does have such positive control. Instead, the problem is that the we-mode group lacks group-level negative control over changing their intention; it is not decidedly *up to the we-mode-group* whether they change their group intention. Even if the majority of members of the pro-life lobby are now collectively committed to advocating pro-choice, if a minority but still substantial set of members sticks to the initial ethos of the group, then, given Closest Continuer, *that* group continues to be the pro-life lobby. Given Closest Continuer, this means that every minority set of members in the we-mode group is akin to the evil neuroscientist in Manipulation. Every minority set of members has the capacity to arbitrarily decisively interfere with the pro-life lobby updating its goal-seeking state, even though no member of the pro-life lobby agreed that they have this kind of authority, simply because they can stick to the initial ethos. The reason-responsiveness of the we-mode group agent is dictated by the reason-responsiveness of various minority sets of members simply in virtue of how such groups are constituted. Because of this, the pro-life lobby lacks negative control over changing their goal-seeking states.

This point generalizes and gives rise to a distinctive problem for purposive we-mode groups as potential duty-bearers, because they may regularly be morally required to do something that stands in conflict with their existing ethos. For example, a riot mob ought to stop rioting and clean up their mess instead. A group of marathon runners ought to stop running the marathon and save someone stuck under a heavy tree instead. And so on. For any substantial change in the ethos, a minority can stick to the current ethos. Note that what is morally required of the group need not necessarily conflict with the ethos. It can simply be a token-goal that is not required for the pursual of the current ethos. The same problem persists. What we can learn from this is that groups that lack the group-level authority to redirect its present goal-seeking states to novel goal-seeking states lack negative control over updating their goal-seeking states.

Thus, the Update Argument is applicable to purposive we-mode group agents in general. In virtue of how they are constituted, purposive groups necessarily lack negative control over updating their goal-seeking states. Even if we were to consider purposive groups as duty-bearers with a limited set of duties, for any intention to perform an action that is currently not required by the ethos, the group lacks negative control. Therefore, purposive groups as we-mode group agents necessarily lack moral competence and do not qualify as duty-bearers.

On both plural subject collectivism and we-mode collectivism, due to the essential features of these accounts that make group intentions non-reductive, these purposive groups lack negative control over changing their intentions.^18^ Their unity and identity over time is intimately tied to the constituting joint or collective commitment to one or more core identity-defining goals, beliefs or values. But this gives (smaller groups of) members the capacity to arbitrarily decisively interfere with any changes to the group’s goal-seeking states.

## Conclusion

If what I have argued here is correct, then one upshot is that regardless of one’s account of group agency, we can exclude certain groups from qualifying as duty-bearers. If moral competence as set out here is necessary for qualifying as a duty-bearer, then purposive groups cannot have group-level duties. We can understand installing a decision-procedure within a group as a way for the group to acquire more group-level negative control over their action-taking, because a collectively accepted decision-making procedure provides the group with group-level authority to redirect its present goal-seeking states to novel goal-seeking states. By changing how the group is constituted, the capacity of members to arbitrarily decisively interfere is removed. In my view, this is the cut-off point for moral competence of group agents. Organized groups may qualify as duty-bearers, purposive groups cannot.

Another upshot of my discussion is that it both strengthens the importance of the debate concerning collective duties and delineates more clearly its scope (Aas, 2015; Björnsson, 2014; Collins, 2019; Goodin, 2012; Lawford-Smith, 2015; Pinkert, 2014; Schwenkenbecher, 2021; Wringe, 2010). There is a clear divide between various types of groups. Only organized groups with an accepted decision-making procedure (potentially) qualify as duty-bearers. Purposive groups (e.g., a riot mob, the pro-life lobby, a trio walking together) like other unorganized groups (e.g., humanity, a random collection of bystanders) fail to qualify as duty-bearers. An adequate account of collective duties must be able to explain the ‘oughts’ that seem to befall upon these groups without making these groups the bearer of a group-level duty (cf. Wringe 2010).

As both Gilbert and Tuomela and Mäkelä argue for group responsibility, one might wonder what this means for the moral responsibility of purposive groups. In short, this depends. On a standard view of exemptions (Wallace, 1994), given that these purposive groups lack moral competence, quite similar to how we exempt young children from moral responsibility because they lack moral competence, we must likewise exempt these agents from moral responsibility. This would make it incoherent to attribute moral responsibility to groups that are not moral agents. Moreover, the (implicit) default view in the literature is that there must be a kind of symmetry between one’s duty-analysis and responsibility-analysis in collective contexts (Lawford-Smith, 2015). If an agent is morally responsible at the group-level, then there must have likewise been a violation of a group-level duty. If one accepts symmetry, then, unless one can show that these purposive groups can have group-level duties in some other way (that is, not in virtue of qualifying as a collective moral agent), it follows that purposive groups cannot be collectively morally responsible at the group-level.

However, another option is to reject the standard view concerning exemptions in collective contexts and to reject symmetry. In my view, even non-agential groups can be collectively morally responsible in a non-distributive way for actions or outcomes (de Haan, 2021a). Elsewhere, I argue that to accommodate our considered moral judgments concerning the (non-distributive) collective responsibility of non-agential groups we must let go of a strict moral agency condition on moral responsibility and replace this with a wrongdoing condition (de Haan, 2021b). What matters is that the social object we attribute moral responsibility to is the originator of moral wrongdoing. Non-agential groups and purposive groups can both commit moral wrongdoing that is irreducibly collective. But I don’t think that we must postulate a group-level duty to explain this. We can explain these ‘oughts’ either in terms of individual duties or shared duties, which are nothing but a conjunction of individual duties that are conditional on each other. While the group does not qualify as a duty-bearer, when members violate their individual duties, this can result in irreducibly collective moral wrongdoing for which we can only hold the non-agential group morally responsible as such. Non-agential groups and purposive groups can be collectively responsible, but such groups cannot bear group-level duties because these groups are not moral agents. Thus, what I have in mind is an ethical theory that is asymmetrical in terms of its duty- and responsibility-analysis in collective contexts. The upshot of such a theory is that we can hold on to the Moral Agency Principle: only moral agents can have moral duties. This is important to make sense of the enabling conditions of the normative reasons that generate such duties. I hope to develop this theory of collective ethics in more detail in the future.

## Footnotes

1 For a discussion of procedural collectivism and the fitness of group agents as duty-bearers, see Collins (2019). For a discussion of procedural collectivism and moral competence, see de Haan (2023) and Hindriks (2018).

2 Wringe (2016) introduces these helpful terms and claims that collective duties of non-agential groups are analytically and ontically non-reductive.

3 R. Jay Wallace (1994) calls this reflective self-control.

4 This is not the only argument in favor of ought-implies-can, but I do think it is an important argument in favor of accepting (some variant of) ought-implies-can as a foundational principle of ethical theory.

5 I am thankful to an anonymous referee for pressing me on this.

6 Some may wonder what this means for Frankfurt-style cases of manipulation (Frankfurt, 1969). Frankfurt attempts to falsify the Principle of Alternative Possibilities (PAP): A person is morally responsible for what he has done only if he could have done otherwise. It is possible to construct Frankfurt-style cases involving local manipulation, that is, the lack of negative control over *one* intentional state. Perhaps this is sufficient to falsify PAP as formulated because it concerns only one action. One could perhaps argue that having negative control over the intention to perform a specific action is not necessary for moral responsibility for *that* action, provided that the agent has sufficient negative control in general. What I am arguing is that if someone else can decisively arbitrarily interfere with *all* (or at least a very large set) of an agent’s intentional states, then that agent is not sufficiently morally competent, because it is not *up to the agent* whether she appropriately responds to reasons. Let me further stress that I think that the reason-responsiveness of a global manipulator makes the case significantly disanalogous to causal determinism.

7 Premise 2 specifies merely a necessary condition for control and not a sufficient one because the agent also needs to have sufficient positive control over her *actions*.

8 Elsewhere, I argue that we need not postulate a group-level duty in order to explain why purposive groups can commit irreducibly collective moral wrongdoing (de Haan, 2021b).

9 (WMI) Agent A has the intention with the collective content P in the we- mode in a group, g, of agents if. (1) A is functioning qua member of g, (2) A’s intention presupposes that the agents in g collectively accept P as their intention (content) for satisfying the interests of g, (3) A intends to participate in the satisfaction of the intention for g, and. (4) A presupposes that the central we-mode criteria are satisfied for the participants. (Tuomela, 2013, 68).

10 For all conditions, see (Tuomela & Mäkelä, 2016, 315).

11 The exact nature of this group-level ‘ought’ is unclear. They say nothing about the reasons that generate such ‘oughts’. Given that Tuomela does hold that there are *group agent* motivating reasons, reasons a group agent has for its action (as distinct from group-based reasons of members), it seems sensible to interpret this as claiming that a *group agent’s* normative reason generates a group ought.

12 Hindriks (2018) argues that a group agent has moral understanding only if it has an effective moral perspective, that is, a set of collectively accepted moral policies. Elsewhere I argue that Hindriks’ view is too stringent.

13 See Collins (2019, 57) for a similar argument about making new decisions. Gunnar Björnsson (2020) objects that the ability to make a decision is not necessary in order to discharge a duty; more often than not, most negative duties are discharged without making any conscious decision. My argument avoids this objection.

14 To the best of my knowledge, Gilbert does not discuss group identity over time anywhere.

15 I’m thankful to an anonymous referee for suggesting this possible response.

16 I slightly changed the notation of time slices. Tuomela loosely bases his account on Kay Mathiesen’s (2003) account. Mathiesen likewise discusses Robert Nozick’s (1981) closest continuer condition but does not (fully) incorporate this into her account.

17 If (i) refers to the collective acceptance of *all* members of g (rather than g’), the account fails. If a small group secedes and adopts a somewhat different ethos, while the remaining members continue to collectively accept the old ethos, then *neither* group would continue to be g (as condition (i) would not be met due to a lack of collective acceptance of *all* members). Clearly, this is false. If (i) refers to the collective acceptance of a *subset* of members of g, the same problem as below exists.

18 I think this conclusion follows for any account of group agency that includes groups that lack the group-level authority to redirect their goal-seeking states. For example, the same problem arises for what Tracey Isaacs (2011) calls goal-oriented collectives, which lack an organizational structure. She argues that goal-oriented collectives, just as organizations, qualify as collective agents, because their collective intentions are irreducible. But note that what exactly counts as an organized group agent may differ too. For example, Collins (2019) and Lawford-Smith (2015) have a wider conception of what organized groups qualify as a group agent than List and Pettit (2011), yet these groups still have group-level authority to redirect goal-seeking states because members must be committed to a decision-making procedure.
